# Peptidomics Analysis Discloses That Novel Bioactive Peptides Participate in Necrotizing Enterocolitis in a Rat Model

**DOI:** 10.1155/2020/4705149

**Published:** 2020-12-31

**Authors:** Yiwen Liu, Changlin Wang, Renqiang Yu, Jianfeng Fan, Weilai Jin, Yuting Zhu, Yingzuo Shi, Yulei Jing, Xiaolei Wang, Zhengying Li, Jian Zhou, Le Zhang

**Affiliations:** ^1^Department of Neonatology, The Affiliated Wuxi Children's Hospital of Nanjing Medical University, Wuxi, 214023 Jiangsu, China; ^2^Department of Neonatology, The Affiliated Wuxi Maternity and Child Health Care Hospital of Nanjing Medical University, Wuxi, 214002 Jiangsu, China; ^3^Department of Pediatrics Surgery, The Affiliated Wuxi Children's Hospital of Nanjing Medical University, Wuxi, 214023 Jiangsu, China; ^4^Department of Pediatric Laboratory, The Affiliated Wuxi Children's Hospital of Nanjing Medical University, Wuxi, 214023 Jiangsu, China

## Abstract

Necrotizing enterocolitis (NEC) is a common devastating gastrointestinal disease in premature infants, the molecular mechanisms of which have not been fully elucidated. Recently, endogenous peptides have garnered much attention owing to their role in diagnosis and treatment. However, changes in the peptide expression of NEC intestinal tissues remain poorly understood. In the present study, a comparative peptidomics profiling analysis was performed between NEC and control intestinal tissues via liquid chromatography-tandem mass spectrometry (LC-MS). In total, 103 upregulated and 73 downregulated peptides were identified in the intestinal tissues (fold change ≥ 1.5, *p* < 0.05). Bioinformatics analysis revealed that these differentially expressed peptides were significantly associated with NEC pathophysiology, including apoptosis, the TGF-*β* signaling pathway, the Wnt signaling pathway, and the MAPK signaling pathway. Furthermore, two putative peptides could inhibit apoptosis and promote the migration of intestinal epithelial cells induced by lipopolysaccharide; these peptides were derived from the protein domains MT1 and EZRI, respectively. In conclusion, our study revealed that endogenous peptides are involved in the pathophysiologic mechanism of NEC; nevertheless, further exploration is required in this regard.

## 1. Introduction

Necrotizing enterocolitis (NEC) is one of the most common fatal diseases in preterm infants, with an incidence rate of 5–10% in very low birthweight (<1500 g) cases [1]. Some studies suggest that the administration of steroids, immunoglobulins, or *Lactobacillus* can reduce the incidence of NEC in neonates. However, the current treatment strategies predominantly focus on medical stabilization and the prevention of disease progression [2]. The mortality rate may rise to 50% when irreversible necrosis or perforation that requires surgery is present [3]. It is known that the disease progresses primarily via the invasion of inflammatory factors and bacteria in the gastrointestinal tract; however, the underlying molecular mechanisms remain to be elucidated completely [4].

Peptidomics, a novel branch of proteomics, has garnered considerable attention for its role as biological markers and therapies [5]. Recently, peptidomics analyses have been applied in detecting peptide changes in different diseases, including cancer [6, 7]. Increasing evidence has shown that endogenous peptides play a crucial role in digestive system diseases [8]. For instance, trefoil factor family peptide 3 (TFF3), mainly located in goblet cells, is significantly upregulated in peptic ulcer. TFF3 not only participates in maintaining and repairing mucosal integrity but also promotes the healing of intestinal ischemia-reperfusion injury in weaned rats [9]. Therefore, an analysis of differentially expressed peptides in NEC tissues may provide new insights to explore treatment strategies.

To explore the peptide expression profile of NEC, quantitative liquid chromatography-mass spectrometry (LC-MS) was performed, and the peptide content of rat NEC intestinal tissue (NEC group) and normal control intestinal tissue (Ctrl group) was analyzed. Based on preliminary functional verification, two downregulated peptides derived from the EZRI (EDP1) and MT1 (MDP2) domains showed a significant protective effect in a lipopolysaccharide- (LPS-) induced NEC model in vitro and are worthy of further study.

## 2. Materials and Methods

### 2.1. Ethics Statement

The study was conducted with the approval of the Animal Ethics Committee of Nanjing Medical University (No: IACUC-1807004). To minimize the suffering of the animals used in this research, the procedures followed were in strict compliance with the Guide for the Care and Use of Laboratory Animals.

### 2.2. Establishment of the Animal Model

Eighteen neonatal Sprague–Dawley rats provided by the Animal Core Facility of Nanjing Medical University were randomly assigned to the Ctrl and NEC groups. The rats in the Ctrl group were fed by their mothers as needed; the NEC rat model was established as follows [10]: (1) hypoxia-cold stimulation: asphyxia stress via exposure to 95% nitrogen twice a day for 5 min, followed by exposure to a cold temperature (4°C) for 10 min; (2) formula feeding (ionized water (10 mL) and Esbilac (PetAg) canine milk (5 mL) (2 : 1) mixed with Similac Advance infant formula (Abbott Nutrition) 2 g): the animals received perfused hypertonic milk (0.08–0.1 g/kg) immediately after cold stimulation. Four days for establishing the model, the rats were sacrificed, and intestinal tissue was collected. Intestinal tissue (3 cm) from the terminal ileum was harvested and subjected to NEC model determination and peptide extraction.

### 2.3. Peptide Extraction and Purification

Three pairs of intestinal tissue samples from the Ctrl and NEC groups were ground into powder in liquid nitrogen and fully compatible with denaturing buffers including 8 M urea/100 mM TEAB (pH 8.0), 1 mM PMSF, 2 mM EDTA, and 10 mM DTT. The mixture was sonicated on ice for 10 min and centrifuged at 4°C (12,000 rpm) for 30 min; then, the supernatant was transferred to a fresh centrifuge tube. MWCO (10 kDa) filter tubes (Millipore, Billerica, MA, USA) were used to filter the supernatant. The filter tubes were centrifuged at 4°C (10,000 × *g*); flow-through containing the peptides was collected and purified using a C18 column. Finally, the flow-through was dried under vacuum and stored at -80°C.

### 2.4. TMT Labeling and LC-MS Analysis

Formic acid (0.1%) was used to dissolve the peptide samples, followed by reducing in 10 mM DTT and alkylating with 50 mM iodoacetamide for 30 min. Following desalting and lyophilization, the peptides were labeled with TMT reagent (TMT 6 Relabeling Reagent, Thermo Fisher Scientific, USA). The labeled peptides were mixed and analyzed via LC-MS/MS [11]; an LTQ-Orbitrap Velos mass spectrometer (Thermo Fisher Scientific) was used for this mass spectrometry analysis. In total, 2000 single emission spectra were accumulated from 10 random positions of each sample. Using isotope correction factors recommended by the manufacturer, search for MS data using MASCOT database (Matrix Science, Boston, MA, USA) and SwissProt database. Additionally, to reduce the possibility of erroneous peptide identification, only those peptides with a significance score (≥20) within a 99% confidence interval and greater than “identity” were counted via MASCOT probability analysis [12].

### 2.5. Bioinformatics Analysis

The characteristics of the identified peptides were analyzed using ProtParam tool. Presumed functions on peptides and protein–protein interactions were predicted using the UniProt and STRING databases. We used DAVID v6.7 (Database for Annotation, Visualization and Integrated Discovery; https://david-ncifcrf.gov/) for conducting GO and KEGG pathway enrichment analyses to investigate the potential functions of peptides and their precursor proteins.

### 2.6. Hematoxylin-Eosin Staining (H&E Staining)

After sacrifice, intestinal tissue from the rats' terminal ileum was harvested and fixed with 4% paraformaldehyde for 1 d at room temperature. Following dehydration, embedding, sectioning, defatting, rehydration, and H&E staining, the tissue sections were observed under a light microscope (Zeiss, Oberkochen, Germany).

### 2.7. Cell Culture

IEC-6 enterocytes were purchased from the American Type Culture Collection (ATCC, USA). The growth medium consisted of high glucose Dulbecco's Modified Eagle's medium (DMEM, Gibco BRL), 10% fetal bovine serum (FBS, Gibco BRL), 1% penicillin-streptomycin solution, and 0.1 U/mL recombinant human insulin. The cells were incubated at 37°C under 5% CO_2_ and treated with 100 *μ*g/mL LPS for 6 h to establish the NEC model in vitro.

### 2.8. Wound-Healing Assay

Cell migration was analyzed using the wound-healing assay. IEC-6 cells were seeded in 6-well plates and cultured in DMEM containing 10% FBS. On reaching confluency, the cell monolayer was scraped using sterile 1 mL pipette tips to form a uniform cell-free area. The wells were gently washed with sterile phosphate-buffered saline (PBS) to remove debris from the medium and monitored under an inverted microscope immediately (Eclipse TS100-F, Nikon) (0 h). Next, fresh medium was added to the wells, and the plates were incubated in the presence of EDP1 or MDP2 (20 *μ*m/L) at 37°C for 12 h and monitored under an inverted microscope (12 h). Images taken at the 0 h and 12 h timepoints were expressed as the migration area, and the distances were measured using the ImageJ software v4.16.

### 2.9. Detection of Apoptosis

According to the manufacturer's instructions, an annexin V-fluorescein isothiocyanate (FITC)/propidium iodide (PI) cell apoptosis kit was used to detect the apoptosis level of the IEC-6 cells. In brief, after incubating the NEC in vitro model with EDP1 and MDP2 for 24 h, the IEC-6 cells were collected and washed with PBS, resuspended in 300 *μ*L binding buffer at a concentration of 1 × 10^6^/mL, and mixed with 3 *μ*L annexin V-FITC and 3 *μ*L PI for 15 min. A BD LSRII Flow Cytometer System with the FACS Diva Software was used to analyze the mixtures within 1 h.

### 2.10. Statistical Analysis

All statistical analyses were performed using the SPSS statistical software. Student's *t*-test was used to assess significant differences between the two groups. Statistical comparisons between multiple groups were analyzed using ANOVA. Statistical significance was determined as *p* < 0.05.

## 3. Results

### 3.1. Establishment of the NEC Model

**Figure 1:**
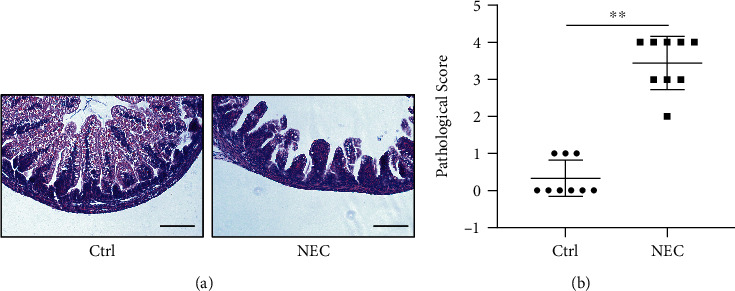
Establishment of the NEC model: (a) H&E staining of representative intestinal tissue sections from the Ctrl and NEC groups (bar = 100 *μ*m); (b) pathological scores of representative intestinal tissue sections from the Ctrl and NEC groups (^∗∗∗^*p* < 0.001).

Hypoxia-cold stimulation combined with hypertonic milk gavage was used to establish the NEC rat model. An optical microscope was used to observe stained intestinal tissue sections and evaluate the pathological score. The histopathological results showed shedding of the intestinal villi, submucosal edema, and inflammatory cell infiltration in the NEC group. In contrast, the intestinal mucosa epithelium was intact, the glands were arranged regularly, and edema and inflammatory cell infiltration were not evident in all layers of the intestinal wall in the Ctrl group (Figure 1(a)). In the NEC rat pups, the pathological score [13] was significantly higher than that in the Ctrl rat pups (Figure 1(b)). These findings revealed that the NEC model was successfully established.

### 3.2. Differentially Expressed Peptides in Intestinal Tissue Samples

**Figure 2:**
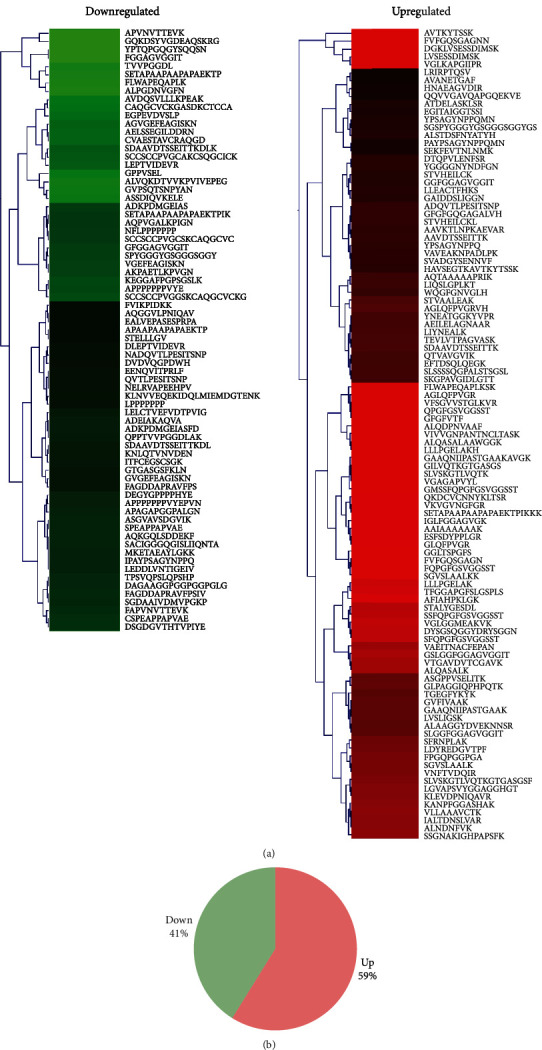
Differentially expressed intestinal peptides between Ctrl and NEC groups: (a) hierarchical cluster analysis of the differentially expressed peptides; (b) number of upregulated or downregulated peptides. The red color represents upregulation, and the green color represents downregulation; a darker color indicates a higher fold change.

TMT-labeled LC-MS/MS analysis was performed on peptides in the intestinal tissue samples from both the Ctrl and NEC groups. A total of 480 nonredundant peptides were identified, with 176 differentially expressed peptides derived from 91 protein precursors (fold change ≥ 1.5, *p* < 0.05). Detailed information on these peptides is shown in Table 1. Upregulated or downregulated differentially expressed peptides were visualized in a hierarchical clustering heat map (Figure 2(a)). The number of upregulated or downregulated differentially expressed peptides is shown in Figure 2(b).

### 3.3. Characteristics of the Differentially Expressed Peptides

**Figure 3:**
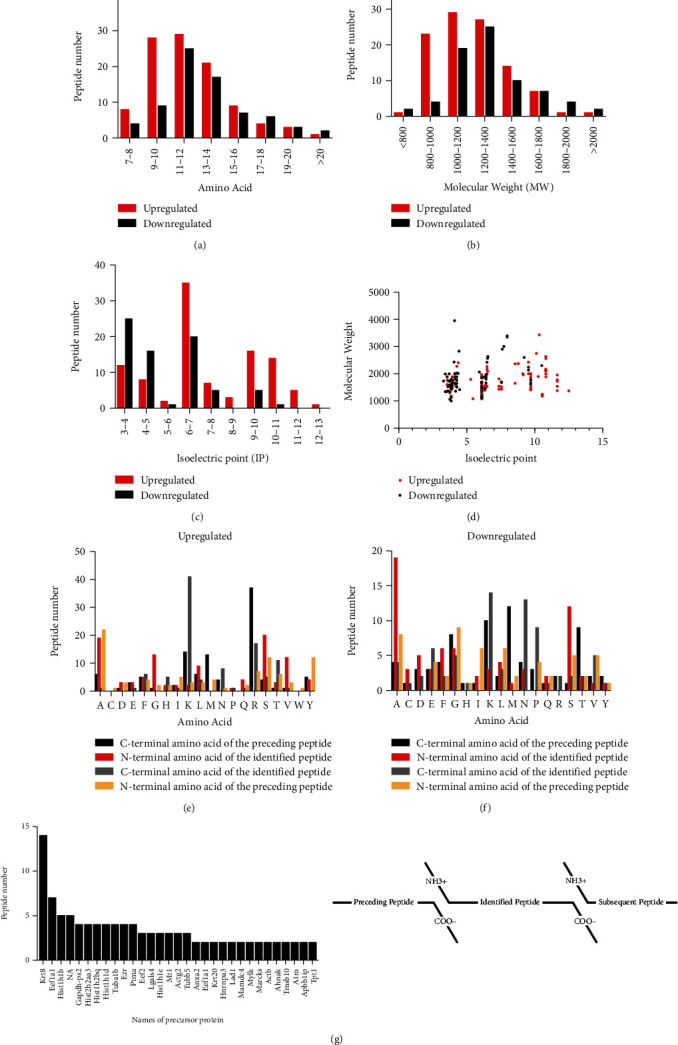
Characteristics of the differentially expressed peptides: (a) amino acid number, (b) molecular weights (MWs), and (c) isoelectric points (pI) of the peptides. (d) Scatter plot of MW versus pI. (e) The distribution diagram of the N- and C-terminal cleavage sites of the upregulated peptides. (f) The distribution diagram of the N- and C-terminal cleavage sites of the downregulated peptides. (g) The number of peptides produced by the same precursor proteins.

Some specific characteristics of the peptides were detected in this study. Among these characteristics, the amino acid numbers are mainly focused on 9–14 (Figure 3(a)), the peptides range largely from 800–1800 (Figure 3(b)), and the pI values are evenly distributed on both sides of acid and basic (Figure 3(c)). In addition, the distribution of MW relative to pI (MW/pI) was investigated based on the amino acid composition and MW distribution specific to a pI (Figure 3(d)). As the generation of peptides mainly depends upon the enzymatic cleavage of precursor proteins [6], the cleavage site patterns of the identified peptides were analyzed (Figures 3(e) and 3(f)). Among the upregulated peptides, lysine (K), arginine (R), alanine (A), and serine (S) were the four dominant amino acids, whereas among the downregulated peptides, alanine (A), lysine (K), asparagine (N), and glutamic acid (E) were the four dominant amino acids. In particular, we found that multiple peptides are derived from same precursor proteins. Among these, the precursor proteins Krt8, Eef1a1, Hist1h1b, and NA generated a total of 31 peptides (Figure 3(g)).

### 3.4. GO and KEGG Pathway Analyses of the Differently Expressed Peptides

**Figure 4:**
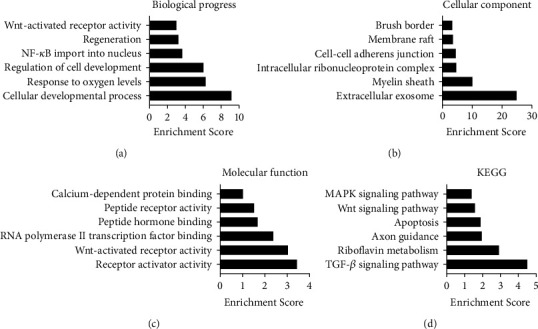
GO and KEGG pathway analyses of the differentially expressed peptides: (a) the biological process categories; (b) the cellular component categories; (c) the molecular function categories; (d) the KEGG signaling pathways.

GO and KEGG pathway analyses were performed to determine the potential roles of these differentially expressed peptides and their corresponding precursor proteins. For biological processes, cellular developmental process, response to oxygen levels, regulation of cell development, NF-*κ*B import into the nucleus, regeneration, and Wnt-activated receptor activity had a high enrichment score (Figure 4(a)). Extracellular exosome, myelin sheath, intracellular ribonucleoprotein complex, cell–cell adherens junction, membrane raft, and brush border as cellular components were found to be closely related to the identified peptides (Figure 4(b)). Regarding molecular function, Wnt-activated receptor activity, RNA polymerase II transcription factor binding, peptide hormone binding, peptide receptor activity, and calcium-dependent protein binding were identified as the most enriched subcategories (Figure 4(c)). Further, KEGG pathway analysis indicated that the potential function of these differentially expressed peptides was related to the TGF-*β* signaling pathway, riboflavin metabolism, axon guidance, apoptosis, the Wnt signaling pathway, and the MAPK signaling pathway (Figure 4(d)).

### 3.5. Bioactive Peptides Potentially Related to NEC

The functions of the precursor proteins largely determine the functions of the bioactive peptides [14]. We used the UniProt and STRING databases to analyze the protein interactions of the differentially expressed peptides and the protein–protein interaction of their protein precursors. It is well known that bioactive peptides can be derived from their precursor domain. Hence, we used the UniProt database to analyze peptides derived from their precursor protein functional domain (Table 2). Among the downregulated peptides, the protein precursor Ezrin (Ezr) plays a vital role in the apical integrity of the intestinal epithelium, and its severe defects can lead to incomplete villi morphogenesis and neonatal death [15]. In addition, Metallothio (MT1) has a protective effect on intestinal mucosal inflammation. Therefore, we attempted to detect bioactive peptides with potential NEC protective effects from the downregulated peptides.

### 3.6. Protective Effects of EDP1 and MDP2

**Figure 5:**
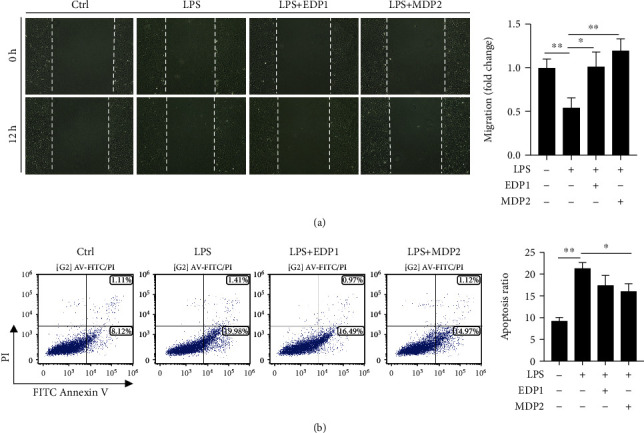
Protective effects of EDP1 and MDP2: (a) the effect of EDP1 and MDP2 on the NEC in vitro model wound restitution; (b) the effect of EDP1 and MDP2 on the NEC in vitro model apoptosis ratio.

To further analyze the function of the differentially expressed peptides, we randomly selected downregulated peptide 1 derived from EZRI (EDP1) and downregulated peptide 2 derived from MT1 (MDP2) to investigate its NEC protective ability. Interestingly, EDP1 treatment could partially reverse the inhibition of cell migration caused by LPS (Figures 5(a) and 5(b)). In addition, MDP2 treatment not only reversed the inhibition of cell migration in the NEC cell model but also decreased the apoptosis level (Figures 5(a) and 5(b)).

## 4. Discussion

NEC is the most common gastrointestinal complication in premature infants [16]. Although extensive research has been conducted to investigate this disease, the underlying molecular mechanisms have not yet been fully elucidated. Peptidomics is an innovative branch of proteomics, which has been used to explore the mechanisms of various diseases [17, 18]. In our study, we detected differentially expressed peptides in intestinal tissues that were further analyzed for their potential association with NEC. Moreover, the bioactive peptides EDP1 and MDP2 showed a protective effect in the NEC model in vitro.

In total, 176 differentially expressed peptides, including 103 upregulated and 73 downregulated, were identified. GO analysis of precursor proteins revealed that the differentially expressed peptides were mainly involved in the response to oxygen levels, NF-*κ*B import into the nucleus, extracellular exosome, intracellular ribonucleoprotein complex, cell–cell adherens junction, peptide hormone binding, and peptide receptor activity. Among these, accumulation of oxygen free radicals and low antioxidant capacity result in intestinal epithelial cell apoptosis and intestinal inflammation, which are the known causes of NEC [19]. Moreover, the aberrant activation of NF-*κ*B has been shown to be associated with many inflammatory bowel diseases, including NEC, and the inhibition of NF-*κ*B can reduce intestinal damage [20]. Further, exosomes partially assumed the function of the local and long-distance communication of cells. These results revealed that the differentially expressed peptides are involved in the pathological mechanism of NEC.

KEGG analysis revealed that the TGF-*β* signaling pathway, apoptosis, and the Wnt signaling pathway had a high enrichment score. It is well known that the main feature of NEC is hemorrhagic and necrotizing inflammation within all layers of the intestinal wall [21]. As a pleiotropic cytokine, TGF-*β* can regulate multiple cellular functions and suppress immune responses [22]. In intestinal immunity, TGF-*β* plays a role in suppressing the strong inflammatory response in the intestinal cavity and promotes the construction of immune tolerance, thereby exerting a protective effect in inflammatory bowel diseases [23]. The destruction of the dynamic barrier between the intestinal epithelial layer and the substances in the intestinal cavity is an important process in NEC pathogenesis [24]. Widespread intestinal epithelial cell death is the key mechanism leading to this damage. Studies have reported high levels of intestinal cell apoptosis in the established NEC animal model; moreover, the effect of caspase inhibitors on apoptosis prevents NEC progression. The renewal of intestinal stem cells is a protective response of the intestine to acute injury. Intestinal renewal as well as stem cell maintenance depends mainly on the Wnt/*β*-catenin pathway [25]. Studies have found that Wnt/*β*-catenin signaling decreases during NEC development [26, 27]. This leads to impaired intestinal stem cell activity and poor intestinal regeneration ability. However, the administration of Wnt can maintain intestinal epithelial homeostasis and avoid the intestinal injury observed in NEC [28]. These results indicate that the differentially expressed peptides may be potentially involved in the mechanism underlying NEC.

Recently, an increasing number of peptides have been found to exert a protective effect in intestinal diseases. For instance, vasoactive intestinal peptides (as effective anti-inflammatory agents) exhibited the ability to regulate homeostasis of the intestinal epithelial barrier. It serves a therapeutic role by reducing inflammation and destroying tight junctions in NEC [29]. In addition, a peptide derived from *Porphyra yezoensis* was able to promote IEC-6 cell proliferation through the activation of insulin-like growth factor I receptors [30]. However, there are no reports about the treatment of NEC with endogenous active peptides.

In our study, peptides derived from the precursor proteins EZRI and MT1 were significantly downregulated in the NEC group. We initially synthesized two peptides EDP1 and MDP2 from the functional domains of Ezrin and Metallothio, respectively. We found that the peptide EDP1 promotes cell migration after acting on the NEC model in vitro, whereas the peptide MDP2 promotes cell migration and inhibits apoptosis in the NEC model in vitro. These results indicate that bioactive peptides are involved in the progression of NEC; however, the mechanisms underlying the peptide functions need to be further investigated.

## 5. Conclusion

In conclusion, we initially screened the differentially expressed peptides in the intestinal tissues of the NEC rat model based on TMT markers combined with LC-MS/MS analysis. Next, we investigated the pathological mechanism of NEC and explored the potential treatment strategies. Although the peptides EDP1 and MDP2 were confirmed to exhibit protective effects in NEC, we need to explore their therapeutic mechanisms further.

## Figures and Tables

**Table 1:** Differential peptides in the intestine tissue of NEC rat model.

Accession	Gene	Peptide	Fold change	*p* value
Upregulated peptides				
G3V8B3	Hist1h2bq	AVTKYTSSK	57.72612	<0.05
P69897	Tubb5	FVFGQSGAGNN	31.84287	<0.01
Q10758	Krt8	DGKLVSESSDIMSK	25.55914	<0.001
Q10758	Krt8	LVSESSDIMSK	22.05736	<0.001
P15999	Atp5a1	VGLKAPGIIPR	21.49717	<0.001
O88989	Mdh1	VIVVGNPANTNCLTASK	12.14385	<0.05
Q66HT1	Aldob	ALQASALAAWGGK	11.93121	<0.001
G3V8B3	Hist1h2bq	LLLPGELAKH	11.86002	<0.001
P04182	Oat	ALQDPNVAAF	11.16052	<0.05
P05197	Eef2	VFSGVVSTGLKVR	10.57569	<0.05
Q10758	Krt8	QPGFGSVGGSST	10.57101	<0.01
F1LNF1	Hnrnpa2b1	GFGFVTF	10.55872	<0.05
K7S2S2	Hist2h2aa3	AGLQFPVGR	10.3368	<0.05
Q7M0E3	Dstn	FLWAPEQAPLKSK	10.01604	<0.01
Q10758	Krt8	GGLTSPGFS	9.126557	<0.001
M0R7B4	Hist1h1d	SGVSLAALKK	8.945185	<0.001
Q10758	Krt8	FQPGFGSVGGSST	8.793551	<0.001
P69897	Tubb5	FVFGQSGAGN	8.771316	<0.05
D3ZGY4	Gapdh-ps2	GAAQNIIPASTGAAKAVGK	8.399639	<0.001
D3ZBN0	Hist1h1b	SLVSKGTLVQTK	8.352781	<0.01
M0R7B4	Hist1h1d	GILVQTKGTGASGS	8.334644	<0.001
P02262	NA	VGAGAPVYL	8.301511	<0.001
Q10758	Krt8	GMSSFQPGFGSVGGSST	8.18755	<0.01
P10719	Atp5b	IGLFGGAGVGK	7.935978	<0.01
B5DEM5	Rpl14	AAIAAAAAAK	7.751116	<0.01
K7S2S2	Hist2h2aa3	GLQFPVGR	7.629358	<0.05
P62630	Eef1a1	ESFSDYPPLGR	7.607978	<0.001
O55159	Epcam	QKDCVCNNYKLTSR	7.485879	<0.001
P15865	Hist1h1e	SETAPAAPAAPAPAEKTPIKKK	7.38236	<0.01
D3ZGY4	Gapdh-ps2	VKVGVNGFGR	7.332894	<0.001
F1M577	LOC100359671	AFIAHPKLGK	6.954795	<0.05
Q6P725	Des	TFGGAPGFSLGSPLS	6.720618	<0.05
G3V8B3	Hist1h2bq	LLLPGELAK	6.595403	<0.01
D3ZIE9	Aldh18a1	VGLGGMEAKVK	6.321181	<0.001
Q925G0	Rbm3	DYSGSQGGYDRYSGGN	6.259407	<0.01
Q10758	Krt8	SFQPGFGSVGGSST	6.237045	<0.01
Q10758	Krt8	SSFQPGFGSVGGSST	6.119025	<0.01
Q9QXQ0	Actn4	STALYGESDL	5.964186	<0.001
Q10758	Krt8	GSLGGFGGAGVGGIT	5.600793	<0.05
P05065	Aldoa	ALQASALK	5.474993	<0.01
M0RA08	Plin3	VTGAVDVTCGAVK	5.466526	<0.001
Q6P9V9	Tuba1b	VAEITNACFEPAN	5.315759	<0.05
A0A0G2K3Z9	NA	SSGNAKIGHPAPSFK	4.969645	<0.01
Q66H80	Arcn1	VLLAAAVCTK	4.865557	<0.05
D3ZGY4	Gapdh-ps2	ALNDNFVK	4.838244	<0.05
B0K031	Rpl7	IALTDNSLVAR	4.834916	<0.001
P62268	Rps23	KANPFGGASHAK	4.801351	<0.001
D3Z7Y6	Krt20	LGVAPSVYGGAGGHGT	4.677876	<0.001
Q10758	Krt8	KLEVDPNIQAVR	4.663074	<0.001
D3ZBN0	Hist1h1b	SLVSKGTLVQTKGTGASGSF	4.551461	<0.01
M0R7B4	Hist1h1d	SGVSLAALK	4.443164	<0.01
P05197	Eef2	VNFTVDQIR	4.417121	<0.01
V5QR27	NA	FPGQPGGPGA	4.341792	<0.05
P63029	Tpt1	LDYREDGVTPF	4.230072	<0.05
Q5EB49	Eno1	SFRNPLAK	4.118444	<0.05
Q80ZE7	Cd2ap	GLPAGGIQPHPQTK	3.871932	<0.01
M0R7B4	Hist1h1d	ASGPPVSELITK	3.803902	<0.001
P18420	Psma1	LVSLIGSK	3.745425	<0.05
P13437	Acaa2	GVFIVAAK	3.727173	<0.01
D3ZGY4	Gapdh-ps2	GAAQNIIPASTGAAK	3.718082	<0.001
Q10758	Krt8	SLGGFGGAGVGGIT	3.670916	<0.001
D3ZBN0	Hist1h1b	ALAAGGYDVEKNNSR	3.654154	<0.05
Q9WVK7	Hadh	TGEGFYKYK	3.578854	<0.01
K7S2S2	Hist2h2aa3	AGLQFPVGRVH	3.386712	<0.05
A0A0G2K6S9	Myh11	STVAALEAK	3.278155	<0.01
P06302	Ptma	SDAAVDTSSEITTK	3.185452	<0.05
G3V779	Lad1	TEVLVTPAGVASK	3.18017	<0.01
B2GVB1	S100a6	LIYNEALK	3.138931	<0.001
K7S2S2	Hist2h2aa3	AEILELAGNAAR	3.121051	<0.01
P69897	Tubb5	YNEATGGKYVPR	3.110143	<0.05
D3Z7Y6	Krt20	SLSSSSQGPALSTSGSL	3.061158	<0.05
P63018	Hspa8	SKGPAVGIDLGTT	3.057092	<0.05
A0A0G2JWK7	Tagln	EFTDSQLQEGK	3.027585	<0.05
P62630	Eef1a1	QTVAVGVIK	3.020094	<0.05
P21913	Sdhb	AQTAAAAAPRIK	2.929567	<0.01
Q5RJK6	Inpp1	LIQSLGPLKT	2.899027	<0.01
P10860	Glud1	VVQGFGNVGLH	2.760697	<0.05
P47875	Csrp1	GFGFGQGAGALVH	2.639933	<0.001
Q63191	Mamdc4	ADQVTLPESITSNP	2.631403	<0.01
Q07936	Anxa2	STVHEILCKL	2.621438	<0.001
P06302	Ptma	AAVDTSSEITTK	2.568546	<0.01
Q3MHS9	Cct6a	AAVKTLNPKAEVAR	2.554117	<0.05
P38552	Lgals4	YPSAGYNPPQ	2.508311	<0.05
P05197	Eef2	VAVEAKNPADLPK	2.48379	<0.01
Q5RKI0	Wdr1	SVADGYSENNVF	2.474195	<0.001
G3V8B3	Hist1h2bq	HAVSEGTKAVTKYTSSK	2.468693	<0.05
Q6URK4	Hnrnpa3	YGGGGNYNDFGN	2.394012	<0.05
Q6VPP3	Clca4	DTQPVLENFSR	2.382552	<0.05
P12346	Tf	LLEACTFHKS	2.333879	<0.05
P63029	Tpt1	GAIDDSLIGGN	2.312302	<0.001
Q10758	Krt8	GGFGGAGVGGIT	2.276436	<0.05
Q07936	Anxa2	STVHEILCK	2.247658	<0.01
P00731	Cpa1	ALSTDSFNYATYH	2.173098	<0.05
Q6URK4	Hnrnpa3	SGSPYGGGYGSGGGSGGYGS	2.128543	<0.05
P38552	Lgals4	YPSAGYNPPQMN	2.11776	<0.001
Q4FZY0	Efhd2	ATDELASKLSR	2.05165	<0.05
A0A0G2QC04	Pls1	EGITAIGGTSSI	2.004625	<0.01
P38552	Lgals4	PAYPSAGYNPPQMN	1.905471	<0.001
Q9Z144	Lgals2	SEKFEVTNLNMK	1.899939	<0.05
D3ZVN3	Fbxo10	HNAEAGVDIR	1.77112	<0.05
G3V779	Lad1	QQVVGAVQAPGQEKVE	1.722176	<0.01
P46462	Vcp	AVANETGAF	1.668905	<0.001
D4A3Z8	Tmco3	LRIRPTQSV	1.649045	<0.05
Downregulated peptides				
A0A0G2K890	Ezr	FVIKPIDKK	-1.50403	<0.05
Q9JJ19	Slc9a3r1	EALVEPASESPRPA	-1.52587	<0.05
K7S2S2	Hist2h2aa3	AQGGVLPNIQAV	-1.53578	<0.05
P15865	Hist1h1e	APAAPAAPAPAEKTP	-1.57197	<0.001
F1M4J0	Rictor	STELLLGV	-1.57785	<0.05
A0A0G2JUA5	Ahnak	DVDVQGPDWH	-1.61943	<0.01
Q63191	Mamdc4	NADQVTLPESITSNP	-1.64595	<0.05
Q6P9V9	Tuba1b	DLEPTVIDEVR	-1.66594	<0.05
B5DFA0	Vil1	EENQVITPRLF	-1.69645	<0.05
A0A0G2K3K2	Actb	NELRVAPEEHPV	-1.70784	<0.05
Q63191	Mamdc4	QVTLPESITSNP	-1.7079	<0.05
D3ZDZ1	Apbb1ip	LPPPPPPP	-1.72276	<0.01
Q5EB49	Eno1	KLNVVEQEKIDQLMIEMDGTENK	-1.7416	<0.05
P62630	Eef1a1	GVGEFEAGISKN	-1.78783	<0.01
P63269	Actg2	FAGDDAPRAVFPS	-1.81415	<0.001
D3ZBN0	Hist1h1b	GTGASGSFKLN	-1.85091	<0.001
D3ZAU0	Muc5b	ITFCEGSCSGK	-1.90446	<0.05
P06302	Ptma	SDAAVDTSSEITTKDL	-1.93122	<0.05
D3ZX87	LOC100910017	KNLQTVNVDEN	-1.93203	<0.05
D3ZUM4	Glb1	LELCTVEFVDTPVIG	-1.97941	<0.01
Q6P9V9	Tuba1b	QPPTVVPGGDLAK	-2.01585	<0.05
P63312	Tmsb10	ADKPDMGEIASFD	-2.02372	<0.001
D4A269	NA	ADEIAKAQVA	-2.02826	<0.001
A0A0G2K890	Ezr	APPPPPPPVYEPVN	-2.10935	<0.01
F1LQ48	Hnrnpl	DEGYGPPPPHYE	-2.13291	<0.01
P08081	Clta	APAGAPGGPALGN	-2.17074	<0.001
P06761	Hspa5	MKETAEAYLGKK	-2.21256	<0.01
Q62803	Spam1	LEDDLVNTIGEIV	-2.21478	<0.05
P38552	Lgals4	IPAYPSAGYNPPQ	-2.21577	<0.01
A0A0G2K6S9	Myh11	AQKGQLSDDEKF	-2.22383	<0.05
P13437	Acaa2	SACIGGGQGISLIIQNTA	-2.23257	<0.01
P30009	Marcks	SPEAPPAPVAE	-2.24701	<0.001
P45592	Cfl1	ASGVAVSDGVIK	-2.24734	<0.001
O55212	NA	DAGAAGGPGGPGGPGLG	-2.30987	<0.05
D4AE06	Fkbp15	TPSVQPSLQPSHP	-2.32989	<0.05
P62630	Eef1a1	SGDAAIVDMVPGKP	-2.36181	<0.001
P63269	Actg2	FAGDDAPRAVFPSIV	-2.37939	<0.05
P62630	Eef1a1	FAPVNVTTEVK	-2.45167	<0.01
P30009	Marcks	CSPEAPPAPVAE	-2.54825	<0.01
A0A0G2K3K2	Actb	DSGDGVTHTVPIYE	-2.55819	<0.01
D3ZFU9	Mylk	AQPVGALKPIGN	-2.77191	<0.05
P15865	Hist1h1e	SETAPAAPAAPAPAEKTPIK	-2.77847	<0.001
Q53Z83	Mt1	SCCSCCPVGCSKCAQGCVC	-2.79709	<0.05
D3ZDZ1	Apbb1ip	NFLPPPPPPP	-2.8025	<0.001
P63312	Tmsb10	ADKPDMGEIAS	-2.84338	<0.001
Q6URK4	Hnrnpa3	SPYGGGYGSGGGSGGY	-2.93019	<0.01
Q10758	Krt8	GFGGAGVGGIT	-2.95692	<0.01
P62630	Eef1a1	VGEFEAGISKN	-2.98559	<0.001
D3ZFU9	Mylk	AKPAETLKPVGN	-2.98675	<0.01
A0A0G2K890	Ezr	APPPPPPPVYE	-3.14377	<0.001
Q91W32	Casp1	KEGGAFPGPSGSLK	-3.15783	<0.001
Q53Z83	Mt1	SCCSCCPVGCSKCAQGCVCKG	-3.27655	<0.01
P06302	Ptma	SDAAVDTSSEITTKDLK	-3.51799	<0.001
B6ID08	Mt2A	SCCSCCPVGCAKCSQGCICK	-3.56818	<0.05
Q6P9V9	Tuba1b	LEPTVIDEVR	-3.6423	<0.001
P62630	Eef1a1	AGVGEFEAGISKN	-3.79477	<0.01
D3ZJF8	Fcgbp	CVAESTAVCRAQGD	-3.88626	<0.001
A0A0G2K890	Ezr	AELSSEGILDDRN	-3.89919	<0.05
A0A0G2JUA5	Ahnak	EGPEVDVSLP	-4.05973	<0.01
Q53Z83	Mt1	CAQGCVCKGASDKCTCCA	-4.23021	<0.01
Q63041	A1m	AVDQSVLLLKPEAK	-4.3155	<0.001
D3ZBN0	Hist1h1b	GPPVSEL	-4.64319	<0.001
P13668	Stmn1	ASSDIQVKELE	-4.88393	<0.01
P97881	Muc13	GVPSQTSNPYAN	-5.02829	<0.001
Q63041	A1m	ALVQKDTVVKPVIVEPEG	-5.23966	<0.01
Q6P9V9	Tuba1b	TVVPGGDL	-5.71525	<0.001
P15865	Hist1h1e	SETAPAAPAAPAPAEKTP	-6.05552	<0.001
Q7M0E3	Dstn	FLWAPEQAPLK	-6.43258	<0.01
P62630	Eef1a1	ALPGDNVGFN	-6.78263	<0.001
Q5PQK2	Fus	YPTQPGQGYSQQSN	-8.52323	<0.05
Q10758	Krt8	FGGAGVGGIT	-8.58147	<0.001
P62630	Eef1a1	APVNVTTEVK	-13.4474	<0.001
P63269	Actg2	GQKDSYVGDEAQSKRG	-15.8726	<0.001

**Table 2:** Differential peptides located in functional domains.

Gene	Accession	Peptide	Peptide domain	*p* value
Hist1h2bq	G3V8B3	AVTKYTSSK	Histone H2B	<0.05
Tubb5	P69897	FVFGQSGAGNN	Tubulin/FtsZ family, GTPase domain	<0.01
Mdh1	O88989	VIVVGNPANTNCLTASK	Ldh_1_N	<0.05
Aldob	Q66HT1	ALQASALAAWGGK	Glycolytic	<0.001
Hist1h2bq	G3V8B3	LLLPGELAKH	Histone H2B	<0.001
Eef2	P05197	VFSGVVSTGLKVR	GTP_EFTU_D2	<0.05
Hnrnpa2b1	F1LNF1	GFGFVTF	RNA recognition motif	<0.05
Hist1h1d	M0R7B4	SGVSLAALKK	Domain in histone families 1 and 5	<0.001
Tubb5	P69897	FVFGQSGAGN	Tubulin/FtsZ family, GTPase domain	<0.05
Gapdh-ps2	D3ZGY4	GAAQNIIPASTGAAKAVGK	Gp_dh_C	<0.001
Hist1h1b	D3ZBN0	SLVSKGTLVQTK	Domain in histone families 1 and 5	<0.01
Hist1h1d	M0R7B4	GILVQTKGTGASGS	Domain in histone families 1 and 5	<0.001
NA	P02262	VGAGAPVYL	Histone 2A	<0.001
Atp5b	P10719	IGLFGGAGVGK	ATPases associated with a variety of cellular activities	<0.01
Gapdh-ps2	D3ZGY4	VKVGVNGFGR	Glyceraldehyde 3-phosphate dehydrogenase, NAD binding domain	<0.001
Hist1h2bq	G3V8B3	LLLPGELAK	Histone H2B	<0.01
Aldh18a1	D3ZIE9	VGLGGMEAKVK	AA_kinase	<0.001
Actn4	Q9QXQ0	STALYGESDL	Ca2+ insensitive EF hand	<0.001
Krt8	Q10758	GSLGGFGGAGVGGIT	Keratin_2_head	<0.05
Aldoa	P05065	ALQASALK	Glycolytic	<0.01
Plin3	M0RA08	VTGAVDVTCGAVK	Perilipin	<0.001
NA	A0A0G2K3Z9	SSGNAKIGHPAPSFK		<0.01
Gapdh-ps2	D3ZGY4	ALNDNFVK	Gp_dh_C	<0.05
Rps23	P62268	KANPFGGASHAK	Ribosom_S12_S23	<0.001
Krt8	Q10758	KLEVDPNIQAVR	Keratin_2_head	<0.001
Hist1h1b	D3ZBN0	SLVSKGTLVQTKGTGASGSF	Domain in histone families 1 and 5	<0.01
Hist1h1d	M0R7B4	SGVSLAALK	Domain in histone families 1 and 5	<0.01
Tpt1	P63029	LDYREDGVTPF	TCTP	<0.05
Eno1	Q5EB49	SFRNPLAK	Enolase, C-terminal TIM barrel domain	<0.05
Psma1	P18420	LVSLIGSK	Proteasome	<0.05
Acaa2	P13437	GVFIVAAK	Thiolase_N	<0.01
Gapdh-ps2	D3ZGY4	GAAQNIIPASTGAAK	Gp_dh_C	<0.001
Krt8	Q10758	SLGGFGGAGVGGIT	Keratin_2_head	<0.001
Hadh	Q9WVK7	TGEGFYKYK	3HCDH	<0.01
Myh11	A0A0G2K6S9	STVAALEAK		<0.01
Ptma	P06302	SDAAVDTSSEITTK	Prothymosin	<0.05
Hist2h2aa3	K7S2S2	AEILELAGNAAR	Histone 2A	<0.01
Tubb5	P69897	YNEATGGKYVPR	Tubulin/FtsZ family, GTPase domain	<0.05
Hspa8	P63018	SKGPAVGIDLGTT	HSP70	<0.05
Eef1a1	P62630	QTVAVGVIK	GTP_EFTU_D3	<0.05
Glud1	P10860	VVQGFGNVGLH	Glutamate/leucine/phenylalanine/valine dehydrogenase	<0.05
Ptma	P06302	AAVDTSSEITTK	Prothymosin	<0.01
Eef2	P05197	VAVEAKNPADLPK	EFG_II	<0.01
Wdr1	Q5RKI0	SVADGYSENNVF	WD40 repeats	<0.001
Hist1h2bq	G3V8B3	HAVSEGTKAVTKYTSSK	Histone H2B	<0.05
Tpt1	P63029	GAIDDSLIGGN	TCTP	<0.001
Krt8	Q10758	GGFGGAGVGGIT	Keratin_2_head	<0.05
Efhd2	Q4FZY0	ATDELASKLSR	Metallothio	<0.05
Pls1	A0A0G2QC04	EGITAIGGTSSI		<0.01
Lgals2	Q9Z144	SEKFEVTNLNMK	Galactoside-binding lectin	<0.05
Fbxo10	D3ZVN3	HNAEAGVDIR	Parallel beta-helix repeats	<0.05
Vcp	P46462	AVANETGAF	ATPases associated with a variety of cellular activities	<0.001
Ezr	A0A0G2K890	FVIKPIDKK		<0.05
Ahnak	A0A0G2JUA5	DVDVQGPDWH		<0.01
Tuba1b	Q6P9V9	DLEPTVIDEVR	Tubulin/FtsZ family, GTPase domain	<0.05
Eef1a1	P62630	GVGEFEAGISKN	GTP_EFTU	<0.01
Ptma	P06302	SDAAVDTSSEITTKDL	Prothymosin	<0.05
Tmsb10	P63312	ADKPDMGEIASFD	Thymosin beta actin-binding motif.	<0.001
Hspa5	P06761	MKETAEAYLGKK	HSP 70	<0.01
Spam1	Q62803	LEDDLVNTIGEIV	Glyco_hydro_56	<0.05
Myh11	A0A0G2K6S9	AQKGQLSDDEKF		<0.05
Acaa2	P13437	SACIGGGQGISLIIQNTA	Thiolase_C	<0.01
Eef1a1	P62630	SGDAAIVDMVPGKP	GTP_EFTU_D3	<0.001
Actg2	P63269	FAGDDAPRAVFPSIV	Actin	<0.05
Actb	A0A0G2K3K2	DSGDGVTHTVPIYE		<0.01
Tmsb10	P63312	ADKPDMGEIAS	Thymosin beta actin-binding motif.	<0.001
Krt8	Q10758	GFGGAGVGGIT	Keratin_2_head	<0.01
Eef1a1	P62630	VGEFEAGISKN	GTP_EFTU	<0.001
Ptma	P06302	SDAAVDTSSEITTKDLK	Prothymosin	<0.001
Tuba1b	Q6P9V9	LEPTVIDEVR	Tubulin/FtsZ family, GTPase domain	<0.001
Eef1a1	P62630	AGVGEFEAGISKN	GTP_EFTU	<0.01
Fcgbp	D3ZJF8	CVAESTAVCRAQGD	von Willebrand factor (vWF) type D domain	<0.001
Ahnak	A0A0G2JUA5	EGPEVDVSLP		<0.01
Mt1	Q53Z83	CAQGCVCKGASDKCTCCA	Metallothio	<0.01
A1m	Q63041	AVDQSVLLLKPEAK	Alpha-2-macroglobulin	<0.001
Stmn1	P13668	ASSDIQVKELE	Stathmin	<0.01
Eef1a1	P62630	ALPGDNVGFN	GTP_EFTU_D2	<0.001
Krt8	Q10758	FGGAGVGGIT	Keratin_2_head	<0.001

## Data Availability

The data that support the findings of this study are available from the corresponding author upon reasonable request.
